# Isolated Unilateral Abducens Nerve Palsy Manifesting as a Rare Complication of Idiopathic Pituitary Apoplexy: A Case Report

**DOI:** 10.7759/cureus.22408

**Published:** 2022-02-20

**Authors:** Salman B Syed, Ahmad A Mourra, Tulika Chatterjee

**Affiliations:** 1 Internal Medicine, University of Illinois College of Medicine at Peoria, Peoria, USA; 2 Internal Medicine, St. Elizabeth Youngstown Hospital, Youngstown, USA

**Keywords:** pituitary emergency, pituitary infarction, abducens nerve palsy, cn6 palsy, cranial nerve 6 palsy, pituitary adenoma, pituitary apoplexy, panhypopituitarism, hypopituitarism

## Abstract

Pituitary apoplexy (PA) is an expansion of a pituitary adenoma due to infarction or hemorrhage of the gland. The term apoplexy usually describes larger bleeds leading to a sudden onset of symptoms. Although it is a rare condition, it can be a life-threatening emergency. PA usually presents with severe headache, nausea, vomiting, visual acuity, and field defects, frequently involving the cranial nerves directly adjacent to the pituitary gland, including third (oculomotor) cranial nerve, fourth (trochlear) cranial nerve, ophthalmic and maxillary branches of the fifth (trigeminal) cranial nerve, and, less commonly, the sixth (abducens) cranial nerve.

Here, we present the case of a 36-year-old male who presented with a one-week history of worsening headache associated with double vision. On physical examination, the patient was noted to have left abducens nerve palsy. MRI brain showed anterior right T1 hyperintensity in the pituitary representing blood products. The patient was treated with analgesics and hormonal therapy with improvement in symptoms and eventual resolution of PA without the need for surgical intervention. PA is an unusual cause of acute isolated abducens nerve palsy which should be identified promptly as it is a life-threatening emergency that can be treated immediately with hormonal replacement followed by a decision to manage conservatively or surgically. The long-term follow-up includes endocrine assessment, visual assessment, and imaging surveillance.

## Introduction

Pituitary tumors comprise approximately 8-15% of all brain tumors [1]. In over 80% of patients, pituitary apoplexy (PA) is the initial presentation of an underlying pituitary tumor [2]. PA is a rare medical emergency and can occur within a normal or adenomatous gland. In the case of an adenoma, rapid tumor growth beyond its blood supply is thought to cause ischemic necrosis, hemorrhage, and sudden enlargement, which may result in the compression of surrounding neural structures, including the hypothalamus [3]. They can cause diverse clinical presentations, generally pressure symptoms and endocrinological abnormalities. The earliest most common symptom of PA is a sudden severe headache which may be accompanied by nausea or vomiting [2]. Cranial nerve dysfunction due to pituitary tumors is thought to occur in 5-17% of pituitary tumor patients, generally manifesting as a dysfunction of the third, fourth, fifth, and sixth cranial nerves, which pass through the cavernous sinus [1]. The oculomotor nerve is the most common cranial nerve affected [1,2,4]. The abducens nerve is least commonly involved, perhaps because, in the cavernous sinus, it is more sheltered from the pituitary expansion than are oculomotor and trochlear nerves [1,3]. Abducens nerve involvement produces horizontal diplopia owing to the inability to abduct the involved eye.

## Case presentation

A 36-year-old male presented to the emergency room with a one-week history of headache and double vision. The patient’s headache was pressure-like, mainly in the occipital region, intermittent, and worsened by light and movement. His headache was associated with double vision and was not relieved by any over-the-counter pain medications. He also complained of loss of libido, dry skin, and weight gain for the past month. On physical examination, he was hemodynamically stable with body temperature, blood pressure, and heart rate of 37.6°C, 116/76 mmHg, and 68 beats per minute, respectively. Cranial nerve examination was notable for the inability to abduct the left eye. Complete blood count and metabolic panel were within normal range.

**Figure 1:**
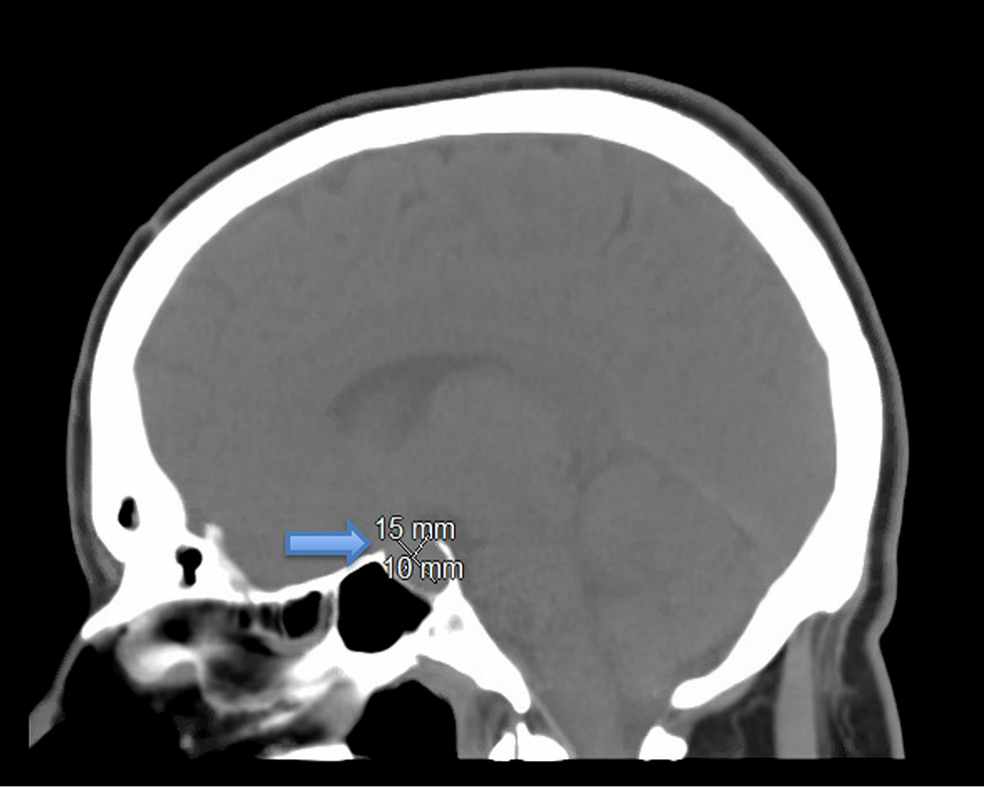
CT of the head without contrast: sagittal view demonstrating prominence of the pituitary gland measuring 1.0 × 1.5 cm with ill-defined hyper-attenuation along the superior aspect. CT: computed tomography

Initial computed tomography (CT) without contrast of the head showed prominence of the pituitary gland measuring 1.0 × 1.5 cm with ill-defined hyper-attenuation along the superior aspect (Figure 1). Lumbar puncture results were negative for meningitis and viral encephalitis, with no elevation of opening pressure.

**Figure 2:**
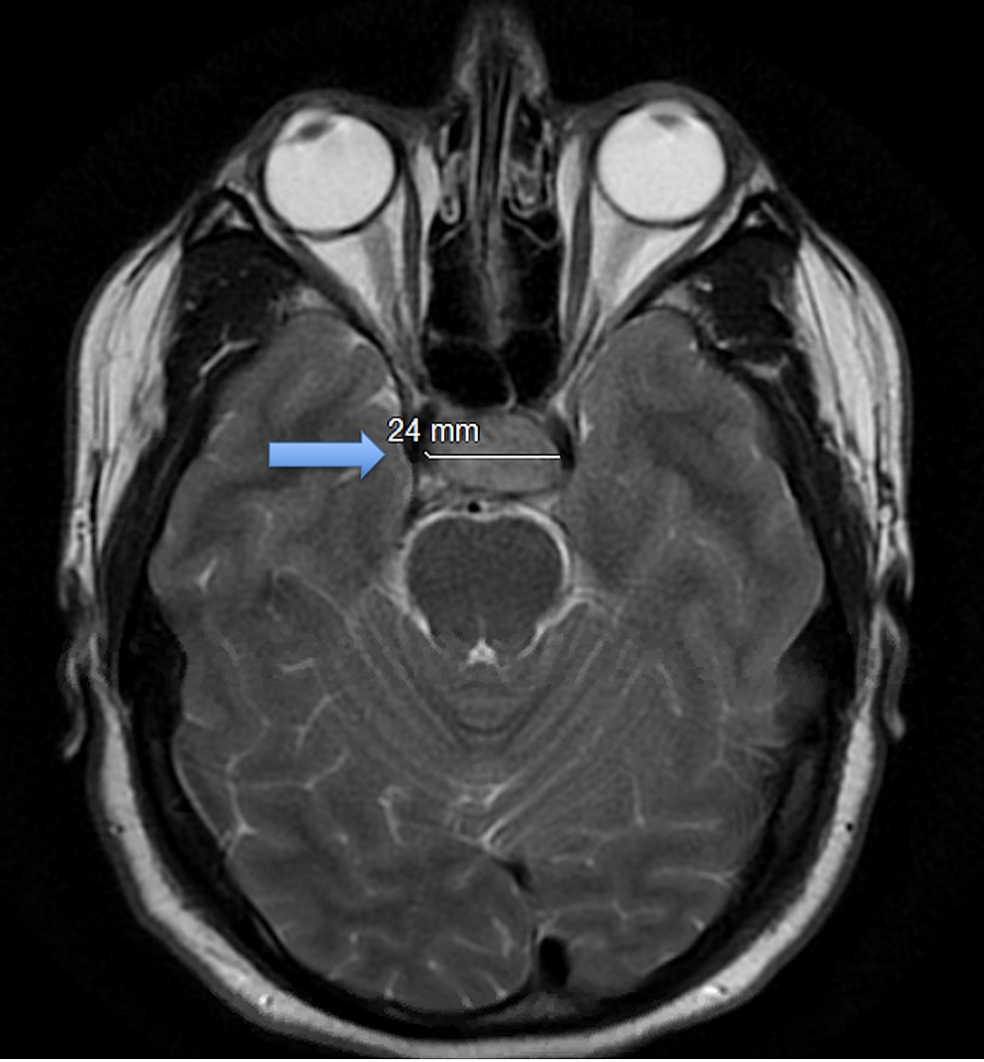
MRI of the brain: axial view demonstrating an area of T1 hyperintensity along the anterior right and lateral aspects of the mass representing blood products consistent with ischemic pituitary apoplexy. MRI: magnetic resonance imaging

Magnetic resonance imaging (MRI) of the brain showed a 1.5 × 2.4 × 1.9 cm complex cystic mass in the pituitary gland representing a cystic adenoma or Rathke’s cleft cyst with bowing of the optic chiasm. An area of T1 hyperintensity was noted along the anterior right and lateral aspects of the mass representing blood products consistent with ischemic PA (Figure 2). Given these findings, hormonal workup was obtained prior to initiating empiric steroid treatment, which included intramuscular injection of hydrocortisone 100 mg followed by hydrocortisone 50 mg every six hours.

Hormonal workup revealed findings consistent with panhypopituitarism (Table 1). Of note, thyroid-stimulating hormone (TSH) level was above the normal range with low T3 and free T4 levels, testosterone levels, luteinizing hormone (LH), follicle-stimulating hormone (FSH), adrenocorticotropic hormone (ACTH), and insulin-like growth factor (IGF) levels were below the normal reference range for age and sex. Although TSH was above the normal range, it was not sufficiently high in the context of low T4 and T3, suggesting an impaired hypothalamic-pituitary-thyroid (HPT) axis. The neurosurgery team recommended outpatient follow-up and a repeat MRI in three to six months to evaluate the need for surgery. An ophthalmological evaluation did not show any other intraocular pathology or changes in vision except for left abducens nerve palsy with diplopia on the left gaze.

**Table 1:** Laboratory workup during the presentation.

Parameter	Value	Normal range
Thyroid-stimulating hormone	5.6 uIU/mL	0.27–4.2 uIU/mL
Triiodothyronine	67.27 ng/dL	80–200 ng/dL
Thyroxine	0.89 ng/dL	0.93–1.7 ng/dL
Testosterone	8 ng/dL	220–1,000 ng/dL
Free testosterone	2.0 pg/mL	47–244 pg/mL
Luteinizing hormone	2.3 mIU/mL	1.7–8.6 mIU/mL
Follicle-stimulating hormone	4.2 mIU/mL	1.5–12.4 mIU/mL
Cortisol	1.08 µg/dL	2.68–18.4 µg/dL
Adrenocorticotropic hormone	6.7 pg/mL	7.2–63.3 pg/mL
Growth hormone	0.36 ng/mL	0.05–3 ng/mL
Insulin-like growth factor	76 ng/mL	83–241 ng/mL

The patient’s headache improved with oral oxycodone-paracetamol 5-325 mg every eight hours, and he was discharged on the fourth day of hospitalization. At the time of discharge, steroid treatment with intravenous (IV) hydrocortisone was switched to dexamethasone 2 mg every six hours for seven days, followed by hydrocortisone 20 mg daily and 10 mg nightly for secondary adrenal insufficiency. Additionally, he was prescribed levothyroxine 150 µg daily for his central hypothyroidism and testosterone 4 mg/24-hour patch daily for his hypogonadotropic hypogonadism. Follow-up appointments with the primary care provider, neurosurgery, and endocrinology services were set up.

Outcome and follow-up

After eight weeks, a repeat MRI brain showed marked improvement with residual 1.0 × 0.5 × 0.9 cm avidly enhancing mass on the left anterior pituitary, which had significantly decreased from the previous 1.5 × 2.4 × 1.9 cm mass, with a resolution of bowing to the optic chiasm. Additionally, a hormonal workup was done to reevaluate the hypothalamic-pituitary-gonadal axis (HPG axis) was obtained. His thyroid hormones were elevated, for which the dose of levothyroxine was adjusted to 125 mg daily based on the free T4 results, no other changes were made. Given this shrinkage in his adenoma and the resolution of the patient’s abducens palsy, the plan was to continue conservative management and hold off on any surgical intervention. At the seven-month follow-up, a repeat MRI showed near-complete resolution of the pituitary adenoma. His levothyroxine dose was reduced to 88 µg daily and he no longer required hydrocortisone and testosterone supplementation based on lab results.

## Discussion

Pituitary adenomas constitute 10% of all intracranial tumors [1]. They are slow-growing tumors and do not have any clinical manifestations until they reach a certain size. PA occurs in 14-22% of patients with adenoma, and clinical findings are manifested in only 0.6-9% of these patients [1]. It is caused by a quick increase in the size of intrasellar contents leading to an increase in the intrasellar pressure [2]. This increase in pressure may lead to the loss of blood supply to the pituitary gland causing infarction, tumor cell death, bleeding, and tumor swelling [2].

The internal carotid artery, oculomotor nerve, trochlear nerve, ophthalmic and maxillary branches of the trigeminal nerve, and the abducens nerve are located near the cavernous sinus. The most affected cranial nerve in PA is the oculomotor nerve followed by the trochlear nerve; however, clinical findings due to the involvement of multiple nerves can be seen [1]. In our patient, isolated abducens nerve palsy developed due to PA, which is extremely rare [1,3,5,6].

Assessment of suspected PA includes a combination of clinical, endocrine, and radiological assessments. Diagnosis of PA should be considered in all patients presenting with acute severe headache with or without neuro-ophthalmic signs [2]. Clinical management includes careful assessment of fluid and electrolyte balance, replacement of corticosteroids, and supportive measures to ensure hemodynamic stability followed by the decision to manage conservatively or surgically [2,4]. In suspected patients with PA, urgent blood samples for endocrine evaluation should be taken prior to administration of steroids. These include random cortisol, prolactin, free thyroxine, TSH, IGF-1, growth hormone, LH, FSH, and testosterone in men and estradiol in women [2]. MRI is the investigation of choice and has been shown to confirm the diagnosis in over 90% of patients [4]. A pituitary CT is indicated if MRI is contraindicated or not possible. The long-term follow-up includes endocrine and visual assessment and imaging surveillance [2].

The major source of mortality associated with PA is acute secondary adrenal insufficiency [2]. In patients with a high index of suspicion for hypoadrenalism or patients who are hemodynamically unstable, prompt IV or intramuscular steroid replacement should be started, as was done in our patient [2,4]. Current treatment protocols are based on guidelines by Baldeweg et al. [2]. Initially, an IV bolus of hydrocortisone 100-200 mg is recommended, followed by either 2-4 mg per hour by continuous infusion or 50-100 mg every six hours by intramuscular injection [2,4]. After the patient has recovered from the acute episode, the hydrocortisone dose can be adjusted to a daily maintenance dosage of 20-30 mg orally usually in two to three divided doses [2]. Surgical intervention is considered in patients with severely reduced visual acuity, severe and persistent visual field defects, and deteriorating levels of consciousness despite steroid replacement and medical stabilization [4]. In our patient, surgery was deferred due to marked clinical improvement with medical management.

Both conservatively and surgically treated patients need close radiological follow-up, and if residual tumor recurrence is detected, additional modalities such as radiotherapy or redo-surgery should be considered [2,4].

## Conclusions

PA is a relatively rare condition. The most affected cranial nerve is the oculomotor nerve; however, abducens nerve palsy may also occur. Isolated abducens nerve palsy is a rare clinical manifestation of PA, and our case highlights that a high index of suspicion is needed for early diagnosis and prompt intervention. Additionally, this case adds to the existing literature of cases reported with this rare presentation.
